# Silencing of A-kinase anchor protein 4 inhibits the metastasis and growth of non-small cell lung cancer

**DOI:** 10.1080/21655979.2021.1977105

**Published:** 2022-03-07

**Authors:** Bo Zhang, Quanteng Hu, Jian Zhang, Zixian Jin, Yuhang Ruan, Lilong Xia, Chunguo Wang

**Affiliations:** a Department of Thoracic Surgery, Taizhou Hospital of Zhejiang Province affiliated to Wenzhou Medical University, Linhai Zhejiang Province China; b Department of Thoracic Surgery, Zhejiang Hospital , No.1229 Gudun Road, Xihu District, Hangzhou Zhejiang province, 310000,China

**Keywords:** AKAP4, non-small cell lung cancer, migration, PKA

## Abstract

Non-small cell lung cancer (NSCLC) is one of the most malignant tumors. The treatment of advanced NSCLC can be challenging due to drug resistance. The discovery of novel cancer-testis antigens to develop new strategies for advanced metastatic NSCLC is required. AKAP4 is an oncogene discovered in some malignant tumors, and its molecular function of AKAP4 in NSCLC is unknown. This study aimed to explore the potential function of AKAP4 in the development and progression of NSCLC. AKAP-4 was found to be significantly upregulated in both clinical NSCLC tissues and NSCLC cell lines. Cell viability and migration were suppressed, apoptosis was induced, and tube formation was inhibited by the knockdown of AKAP-4, accompanied by the downregulation of VEGF, N-cadherin, EphA2, and MMP-2, and upregulation of c-AMP, PKA, and E-cadherin. *In vivo* xenograft experiments revealed that tumor growth was inhibited by the knockdown of AKAP4, accompanied by the activation of c-AMP/PKA signaling and inhibition of epithelial-mesenchymal transition progression. Our results show that AKAP4 might be an important target for treating NSCLC because of its function in promoting the migration and proliferation of NSCLC cells.

## Introduction

Lung cancer is a malignant tumor with the highest morbidity and mortality worldwide, among which non-small cell lung cancer (NSCLC) accounts for approximately 80%-85% of cases. Local diffusion or distant metastasis is commonly observed in patients diagnosed with NSCLC [1]. Due to drug resistance, the treatment of advanced NSCLC using targeted therapies, such as inhibitors or antagonists of epidermal growth factor receptor (EGFR) and anaplastic lymphoma kinase, can be challenging [2]. In addition, it has been reported that EGFR is positive in a relatively small percentage of the white population and that there are population studies showing variations between Asia, Europe, and America [3,4]. Recently, immunotherapy for the treatment of NSCLC has received great attention, including classic immunotherapy and immune checkpoint inhibitors. Tumor vaccines are regarded as the classic immunotherapy. The current tumor vaccines available include whole-cell vaccines (such as dendritic cell vaccines), tumor peptide vaccines (such as L-BLP25), and genetic engineering vaccines (such as Belagenpumatucel-L) [5,6]. Recently, immune checkpoint inhibitors have been widely applied for the treatment of advanced NSCLC and breast cancer, including CTLA-4 and PD-1 inhibitors [7,8]. Although promising clinical progression has been achieved by these immunotherapies, significant adverse effects and drug resistance still arise, which will limit their application [9]. Therefore, there is an urgent need to develop novel heterogeneous immune antigens for the treatment of advanced NSCLC.

Cancer-testis antigens (CTAs) are a group of heterogeneous antigens that are highly expressed in tumor tissues, testicular tissues, and placental tissues and are rarely expressed in other normal tissues [10]. A high correlation between the expression of CTAs and the progression and metastasis of malignant tumors has been widely reported [11]. As human leukocyte antigens are rarely expressed in testicular tissues and placental tissues, mild autoimmune reactions are induced by CTAs, which makes CTAs potentially superior immune targets in the field of immunotherapy [12]. A-kinase anchor protein 4(AKAP4) is a sperm fibrous sheath protein and is reported to be an important regulator of sperm flagellum function [13]. AKAP4 is a type of CTA with low expression in normal tissues, except for testicular and placental tissues. In addition, AKAP4 is a novel CTA that is abnormally expressed in multiple types of malignant tumors, including ovarian cancer [14], cervical cancer [15], multiple myeloma [16], breast cancer [17], and prostate cancer [18]. Gumireddy et al. identified AKAP4 as a potential biomarker for NSCLC by analyzing CTA messenger ribonucleic acids (mRNAs) in peripheral blood monocytes isolated from 116 patients with NSCLC, which was further verified in 264 patients and 135 healthy volunteers. In addition, the expression level of AKAP4 increased significantly with the development of neoplasm staging. In subsequent follow-up investigations, AKAP4 was found to be greatly downregulated in surgical patients and upregulated in relapse patients [19]. However, the molecular function of AKAP4 in the development and progression of NSCLC remains unclear.

In the present study, we suspected that AKAP4 facilitated the metastasis and growth of NSCLC cells by regulating epithelial-mesenchymal transition (EMT) progression and the c-AMP/PKA pathway. The present study aimed to explore the potential therapeutic targets for the treatment of advanced NSCLC by investigating the regulatory mechanism of AKAP4 on the growth and migration of NSCLC.

## Materials and methods

### Collection of clinical tissues and cell treatments

Six patients diagnosed with advanced NSCLC underwent surgery to remove tumor tissues, which were connected with para-carcinoma tissues. Tumor tissues and para-carcinoma tissues were identified and separated by an experienced oncologist who performed the surgeries. The surgeries were conducted in Taizhou Hospital of Zhejiang Province Hospital. The collection of tissues was approved by the ethics committee of Taizhou Hospital of Zhejiang Province Hospital. The NSCLC cell lines, including A549, NCI-H1299, and H460 cells, and the normal human lung epithelial cell line, BEAS-2B, were obtained from ATCC (Maryland, USA) and cultured in Dulbecco’s modified eagle medium (DMEM) supplemented with 10% fetal bovine serum at 5% CO_2_ and 37°C. Human umbilical vein endothelial cells (HUVECs) were purchased from ATCC (Manassas, VA, USA) and cultured in endothelial cell medium at 5% CO_2_ and 37°C.

### Real-time polymerase chain reaction (RT-PCR)

TRIzol© reagent (Invitrogen. California, USA) was used to extract total RNA from the cells in each group, which were further reverse-transcribed to cDNA using the TaqMan miRNA reverse transcription kit (Invitrogen. California, USA). RT-PCR was conducted using SYBR® Green Real‐time PCR Master Mix (Roche Diagnostics, Basel, Switzerland), with the following conditions: 94°C for 5 min, 30 cycles of 94°C for 30 s, and 58–61°C for 30 s, followed by 72°C for 2 min. Glyceraldehyde 3-phosphate dehydrogenase (GAPDH) was used to normalize the relative expression of target genes, which was calculated using the 2^−ΔΔCt^ method [20].

### Western blot assay

Following isolation of the total proteins from the treated cells, a BCA kit (Merck, New Jersey, USA) was used to determine the concentration of proteins, and about 40 μg of protein was loaded, followed by separation with 12% SDS-PAGE. Then, the proteins in the SDS-PAGE were transferred to a polyvinylidene difluoride membrane (Merck, New Jersey, USA). The membrane was subsequently incubated with 5% BSA for 2 h, followed by incubation with primary antibodies against E-cadherin (1:800, Abcam, Cambridge, UK), N-cadherin (1:800, Abcam, Cambridge, UK), PKA (1:800, Abcam, Cambridge, UK), vascular endothelial growth factor (VEGF; 1:800, Abcam, Cambridge, UK), EphA2 (1:800, Abcam, Cambridge, UK), MMP-2 (1:800, Abcam, Cambridge, UK), and GAPDH (1:800, Abcam, Cambridge, UK) overnight. After incubation in a solution of secondary antibody (1:2000, Abcam, Cambridge, UK) for 1.5 h, the membrane was exposed to ECL solution (Invitrogen, California, USA), and the relative expression level of target proteins was confirmed by visualization with Image J software [21].

### Immunohistochemical analysis

Briefly, tissues were fixed with 4% paraformaldehyde, dehydrated, and embedded in paraffin. Subsequently, tissues were cut into 5 μm sections, which were deparaffinized and rehydrated. After incubation in 5% BSA for half an hour, the sections were incubated with primary antibodies against c-AMP (1:200, Abcam, Cambridge, UK), PKA (1:200, Abcam, Cambridge, UK), AKAP4 (1:200, Abcam, Cambridge, UK), or VEGF (1:200, Abcam, Cambridge, UK), followed by incubation with horseradish peroxidase (HRP)-conjugated secondary antibody. Finally, images were taken using a light microscope (Laird, Missouri, USA). Five high-power fields were selected and imaged randomly on each slide, and the average optical density was calculated using ImageJ software [22].

### Transfection

Two independent siRNAs (siRNA 1# and siRNA 2#) were designed to knockdown the expression level of AKAP4. In brief, H460 cells or HUVECs were seeded on 6-well plates at a density of 150,000 cells/well, followed by replacement of the complete culture medium with serum-free and antibiotic-free medium 1 h before transfection. Subsequently, cells were incubated with transfection mixtures containing 30 nM siRNAs using Lipofectamine 3000 (Invitrogen, Carlsbad, USA) according to the manufacturer’s instructions for 5 h, followed by replacement of the medium with the full culture medium. After 24–72 h incubation, cells at approximately 90% confluence were harvested for further analysis. SiNC was used as the negative control. The sequences of the siRNAs were as follows: siNC (5ʹ-ATCTCGCTTGGGCGAGAGTAAG-3ʹ), siRNA #1 (5ʹ-TCTATGTTCACTTGATCGG-3ʹ), and siRNA #2 (5ʹ-CAAGCGAACGGGCAATTTA-3ʹ). qRT-PCR and western blotting assays were used to evaluate the efficacy of the transfection.

### Flow cytometry for the analysis of apoptosis

Cells were seeded on 6-well plates and incubated at 37°C for 48 h, followed by centrifugation at 300 × g for 5 min. The cells were collected and resuspended in serum-free medium and approximately 10 μL Annexin V reagent (eBioscience, California, USA) and 5 μL PI reagents (eBioscience, California, USA), followed by incubation for 10 min at room temperature in the dark. Then, the cell suspension was mixed with phosphate-buffered saline in the flow tube and analyzed by BD flow cytometry (BD, New York) for the analysis of apoptosis [23].

### Tube formation assay

After pre-cooling, the 96-well plate was coated with 50 μL of growth-factor-reduced Matrigel (BD Biosciences), followed by incubation for 30 min. HUVECs treated with different strategies were starved in serum-free medium for 24 h prior to the assay, followed by planting 2 × 10^4^ HUVECs into each well with 200 μL medium and concentrated 75-fold using an ultrafiltration device (Millipore, USA). Tube formation of HUVECs was observed at different time points during a 12-h experimental period using a microscope. The total tube length was measured and analyzed using Image-Pro-Plus 6.0 [24].

### Transwell assay

Approximately 5 × 10^4^ H460 cells were suspended in serum-free DMEM and plated into the upper insert of a 24-well transwell chamber. The cells were then incubated at 37°C for 8 h. The non-migratory cells in the upper layer were removed, and the migratory cells were fixed with 4% paraformaldehyde at room temperature for 10 min, followed by staining with crystal violet solution. Images were photographed under a light microscope (Olympus, Tokyo, Japan) and quantified by counting the number of cells in five randomly selected fields of view for each well [25].

### Enzyme-linked immunosorbent assay (ELISA) for the detection of c-AMP

[26]. A commercial ELISA kit (R&D Systems, Minneapolis, MN, USA) was used to detect the concentration of c-AMP in H460 cells. Briefly, after centrifugation, the supernatant was collected and transferred to 96-well plates, followed by incubation in 1% BSA for the removal of non-specific binding proteins. Then, the primary antibody of c-AMP was added after washing, followed by three washes and incubation with the streptavidin- HRP-conjugated secondary antibody for 30 min. After adding the 3,3ʹ,5,5ʹ-Tetramethylbenzidine solution for 20 min, the reaction was terminated using a stop buffer. Finally, a fluorescence microscope (Leica, Wetzlar, Germany) was used to measure the absorbance at 450 nm.

### Xenograft model

Female Babl/c nude mice (seven to nine weeks old) were purchased from Shanghai Slac. The xenograft models were established by subcutaneously injecting Babl/c nude mice with 5 × 10^5^ cells/0.1 mL H460 cells, H460 cells transfected with siNC, H460 cells transfected with siRNA #1, and H460 cells transfected with siRNA 2# dissolved in 50% Matrigel® (BD Biosciences, New York, USA), respectively (n = 6 for each group). The length (L) and width (W) of each tumor were measured weekly for the calculation of tumor size according to the formula (*L* × *W*^2^)/2. At the end of the animal experiments, tumor tissues were collected and weighed after sacrificing the animals [27].

### Statistical analysis

The data obtained from the present study were expressed as the mean±SD. The data analysis was conducted using GraphPad software. The Student’s t-test was used to analyze data between two groups, and a one-way analysis of variance with the least significant difference test was used to analyze among groups. A P-value of < 0.05 was considered statistically significant.

## Results

We suspected that AKAP4 functions as an oncogene in NSCLC development. The present study aimed to investigate the regulatory mechanism of AKAP4 in the growth and migration of NSCLC cells. The relative expression level of AKAP4 was assessed in both NSCLC tissues and cell lines. Subsequently, we established an AKAP4-knockdown H460 cell line. We further explored the proliferation and migration ability of AKAP4-knockdown H460 cells, as well as the angiogenesis of AKAP4-knockdown HUVECs. In addition, the activity of the cAMP-PKA pathway and EMT progression in AKAP4-knockdown H460 cells was evaluated. Lastly, the growth of AKAP4-knockdown H460 cells in xenograft mice was investigated, along with the activity of the cAMP-PKA pathway and EMT progression in xenograft tumor tissues.

### AKAP4 was highly expressed in NSCLC tissues and cell lines

**Figure 1.**
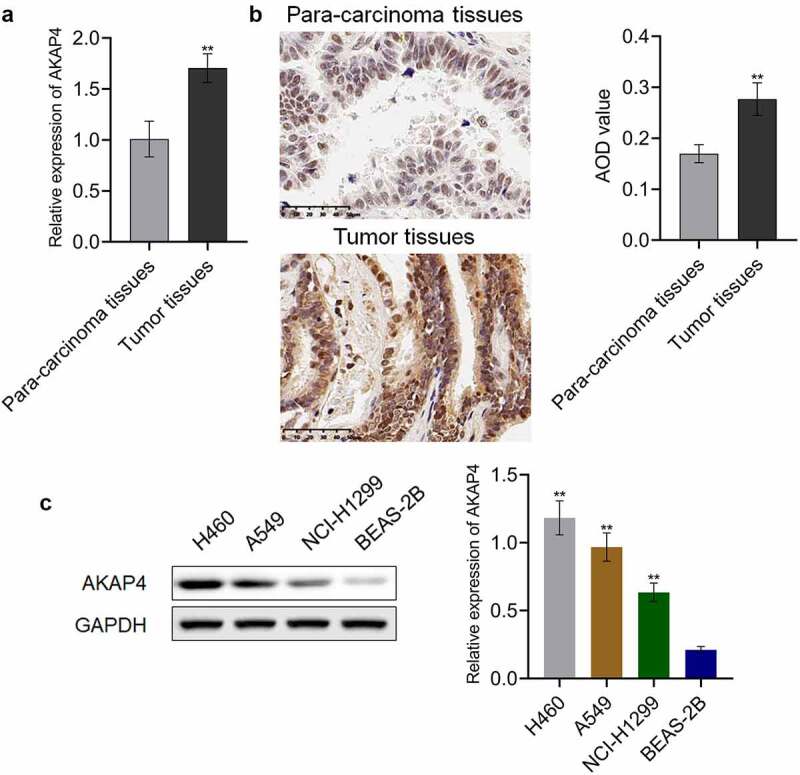
A high expression level of AKAP4 was observed in both clinical NSCLC tissues and NSCLC cell lines. (a). RT-PCR was used to determine the gene expression level of AKAP-4 in the NSCLC tissues and para-carcinoma tissues. (b). Immunohistochemical assay was used to determine the protein expression level of AKAP-4 in the NSCLC tissues and para-carcinoma tissues (**p < 0.01 vs. para-carcinoma tissues). (c). Western blotting assay was used to determine the expression level of AKAP4 in the NSCLC cell line and HEAS-2B cells (**p < 0.01 vs. HEAS-2B cells).

To investigate the role of AKAP4 in the progression of NSCLC, we first measured the expression level of AKAP4 in NSCLC tissues and cell lines. As shown in Figure 1a-b, compared to para-carcinoma tissues, AKAP4 was significantly upregulated in clinical NSCLC tissues (**p < 0.01, vs. para-carcinoma tissues). In addition, compared to the normal human lung epithelial cell line, BEAS-2B cells, the expression level of AKAP4 was dramatically elevated in the NSCLC cell lines (Figure 1c), among which the highest expression level of AKAP4 was observed in H460 cells (**p < 0.01, vs. BEAS-2B cells). Therefore, in subsequent experiments, H460 cells were used as the investigational target in the present study.

### AKAP4 facilitated the proliferation of H460 cells

**Figure 2.**
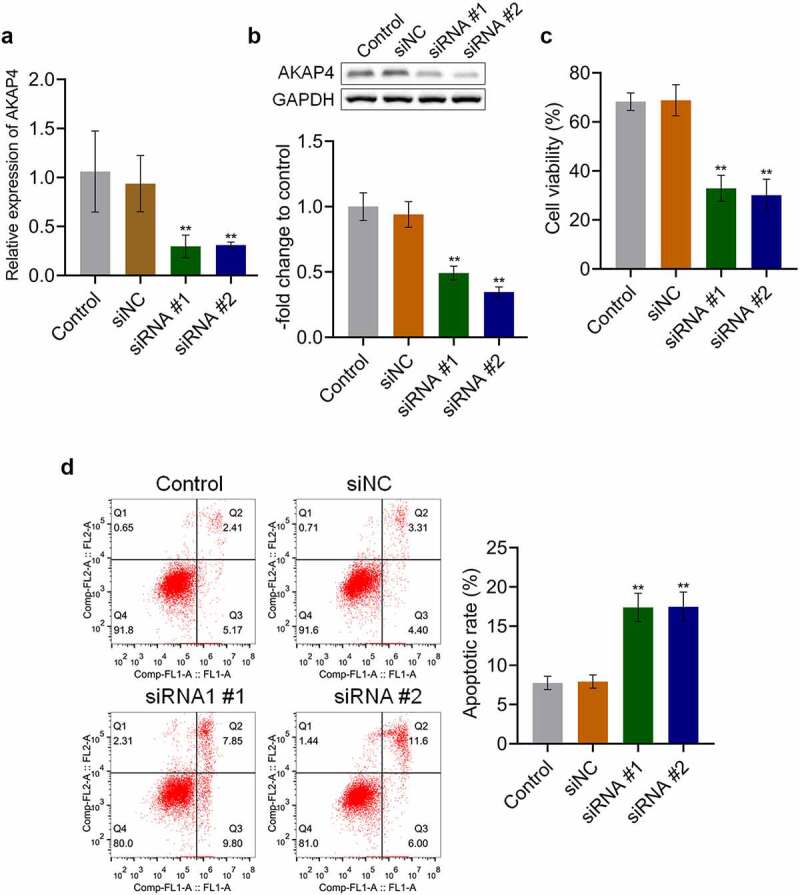
Proliferation was suppressed by the knockdown of AKAP4. (a). The gene expression level of AKAP4 was detected by RT-PCR assay. (b). The protein expression level of AKAP4 was detected by western blotting assay. (c). The cell viability was measured by CCK-8 assay. (d). The apoptosis was determined by flow cytometry (**p < 0.01 vs. siNC). The control was H460 cells incubated with blank completed medium.

To explore the effects of AKAP4 in the progression of NSCLC, H460 cells were transfected with siRNA #1 or siRNA #2 to knockdown the expression level of AKAP4. As shown in Figure 2a-b, the efficacy of siRNAs was verified by RT-PCR and western blotting assays (**p < 0.01, vs. siNC). Then, the proliferation and apoptosis of transfected H460 cells were detected. As shown in Figure 2c, compared to siNC, cell viability was significantly suppressed by the transfection of siRNA #1 and siRNA #2 (**p < 0.01, vs. siNC). In addition, compared to the control group, the apoptotic rate (Figure 2d) slightly changed from 7.58% to 7.71% in the siNC group, which was dramatically elevated to 17.65% and 17.60% after transfection with siRNA #1 and siRNA #2, respectively (**p < 0.01, vs. siNC). These data reveal that proliferation was inhibited, and apoptosis was induced by knocking down the expression level of AKAP4 in H460 cells.

### *AKAP4 induced the migration of H460 cells and angiogenesis* in vitro

**Figure 3.**
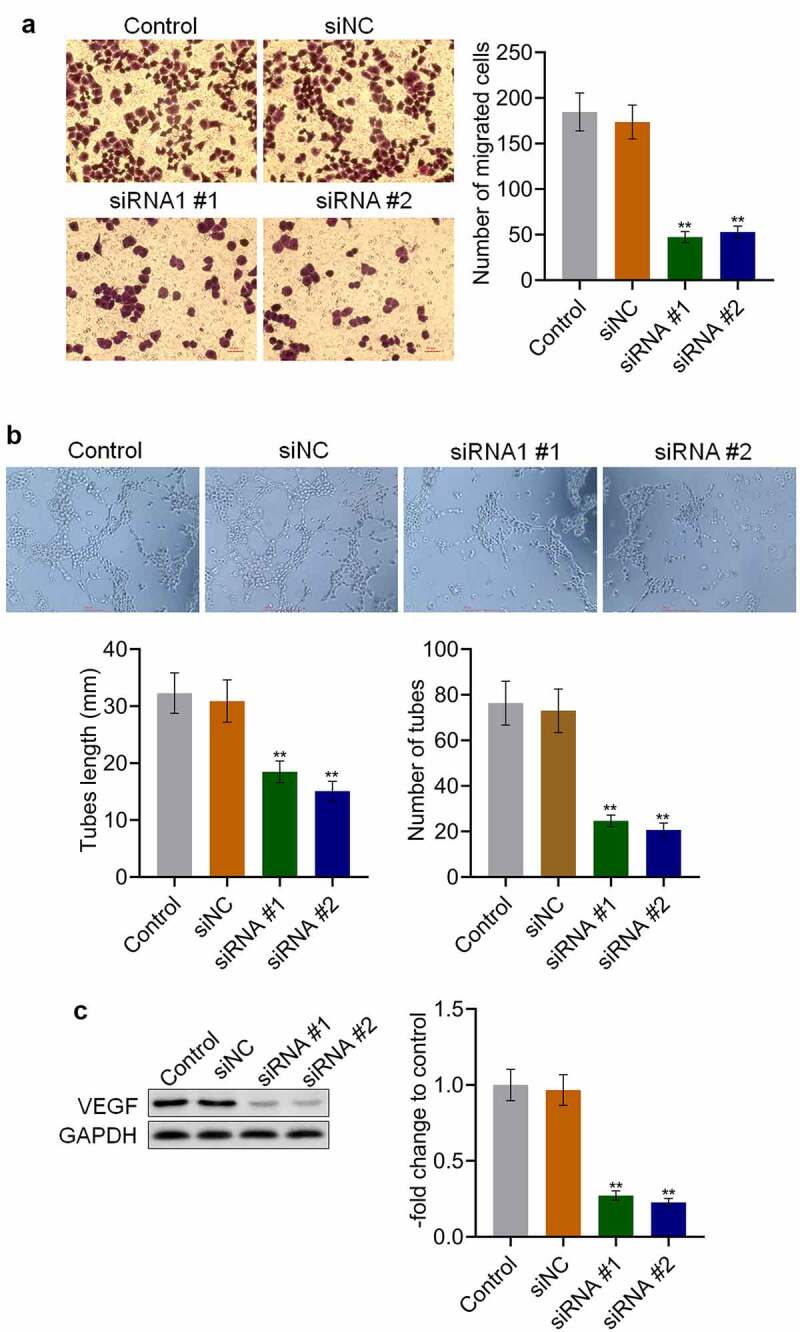
Migration and angiogenesis were inhibited by the knockdown of AKAP4. (a). Transwell assay was used to determine the migration ability of H460 cells. (b). Tube formation assay was used to evaluate the angiogenesis of HUVECs. C. The expression level of VEGF was determined by western blotting assay (**p < 0.01 vs. siNC). The control was H460 cells incubated with blank completed medium.

We further explored the effects of AKAP4 on the metastasis of H460 cells. As shown in Figure 3a, no significant difference was observed in the number of migrated cells between the control and siNC groups, which was significantly declined in siRNA #1 and siRNA #2 transfected H460 cells (**p < 0.01, vs. siNC). The siRNAs were transfected into HUVECs to investigate the effects of AKAP4 on angiogenesis. The tube length was 32.3 mm and 30.89 mm in the control and siNC groups, respectively. Compared to siNC (Figure 3b), the tube length was significantly decreased to 18.5 mm and 15.09 mm in the siRNA #1 and siRNA #2 groups, respectively (**p < 0.01, vs. siNC). In addition, compared to the control group, the tube number was slightly changed from 76 to 73 by transfection with siNC, which was significantly reduced to 25 and 21 by the transfection of siRNA #1 and siRNA #2, respectively (**p < 0.01, vs. siNC). As shown in Figure 3c, compared to siNC, VEGF was found to be significantly downregulated in the siRNA #1 and siRNA #2 groups (**p < 0.01, vs. siNC). These results indicate that the migration of H460 cells and angiogenesis *in vitro* were dramatically alleviated by the knockdown of AKAP4.

### AKAP4 inhibited the c-AMP/PKA pathway and facilitated EMT progression in H460 cells

**Figure 4.**
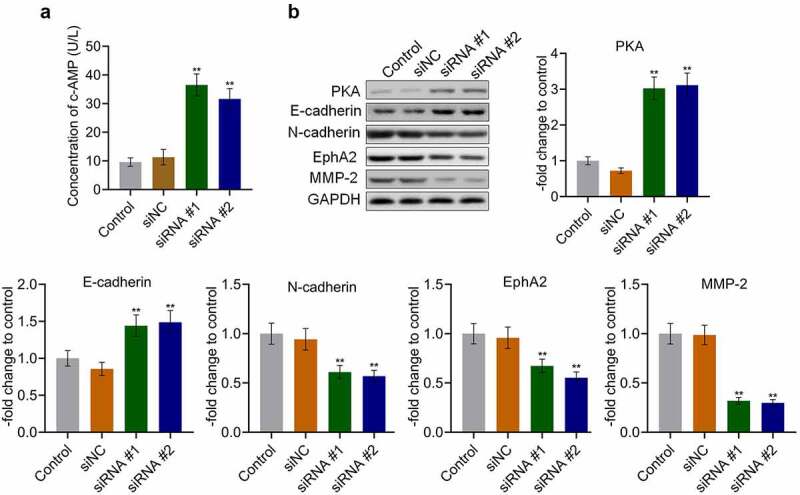
The c-AMP/PKA signaling was activated, and EMT progression was suppressed by the knockdown of AKAP4. (a). The production of c-AMP was measured by ELISA. (b). The expression level of PKA, E-cadherin, N-cadherin, EphA2, and MMP-2 was determined by western blotting assay (**p < 0.01 vs. siNC). The control was H460 cells incubated with blank completed medium.

To explore the potential mechanism underlying the effects of AKAP4 on NSCLC progression, the activity of the c-AMP/PKA pathway and progression of EMT were measured. As shown in Figure 4a, the production of c-AMP was 9.59 U/L and 11.29 U/L in the control and siNC groups, which was dramatically promoted to 36.50 U/L and 31.66 U/L by the transfection of siRNA #1 and siRNA #2, respectively (**p < 0.01, vs. siNC). In addition, compared to siNC, the expression level of PKA (Figure 4b) was markedly elevated in the siRNA #1 and siRNA #2 groups (**p < 0.01, vs. siNC), indicating that the c-AMP/PKA pathway was activated following the knockdown of AKAP4. Compared to siNC, the expression level of the epithelial biomarker E-cadherin was greatly elevated, and the expression levels of the mesenchymal biomarkers N-cadherin, EphA2, and MMP-2 were significantly downregulated by the transfection of siRNA #1 and siRNA #2, respectively (**p < 0.01, vs. siNC). These results reveal the effects of AKAP4 on the c-AMP/PKA pathway and EMT progression.

### *AKAP4 promoted tumor growth in H460 cells and* in vivo *angiogenesis*

**Figure 5.**
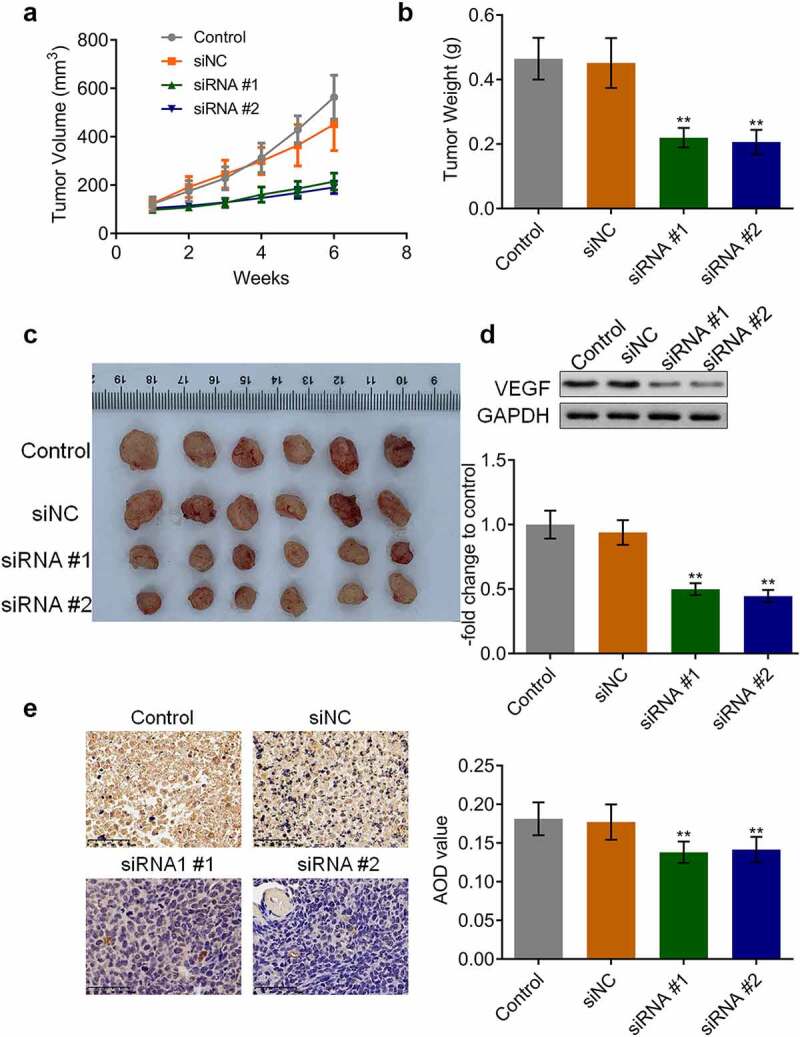
The *in vivo* growth of the tumor was inhibited by the knockdown of AKAP4. (a). Tumor volumes were calculated every week after the planting. (b). Tumor weights were weighed at the end of the experiments. (c). The images of the tumor tissues in each group. (d). The expression level of VEGF was determined by western blotting assay. (e). The expression level of VEGF was determined by immunohistochemical assay (**p < 0.01 vs. siNC). The controls were nude mice planted with H460 cells incubated with blank completed medium.

We further verified the function of AKAP4 in the H460 xenograft model. As shown in Figure 5a-c, after a six-week growth, the tumor volume and weight were significantly reduced in the siRNA #1 and siRNA #2 groups compared to the siNC group, respectively (**p < 0.01, vs. siNC), indicating a promising inhibitory effect by knocking down AKAP4. In addition, the biomarker of angiogenesis, VEGF (Figure 5d-e), was dramatically downregulated in the siRNA #1 and siRNA #2 groups (**p < 0.01, vs. siNC), indicating that the *in vivo* angiogenesis in H460 tumor tissues was suppressed by the knockdown of AKAP4.

### AKAP4 inhibited the c-AMP/PKA pathway and facilitated EMT progression in H460 xenograft tissues

**Figure 6.**
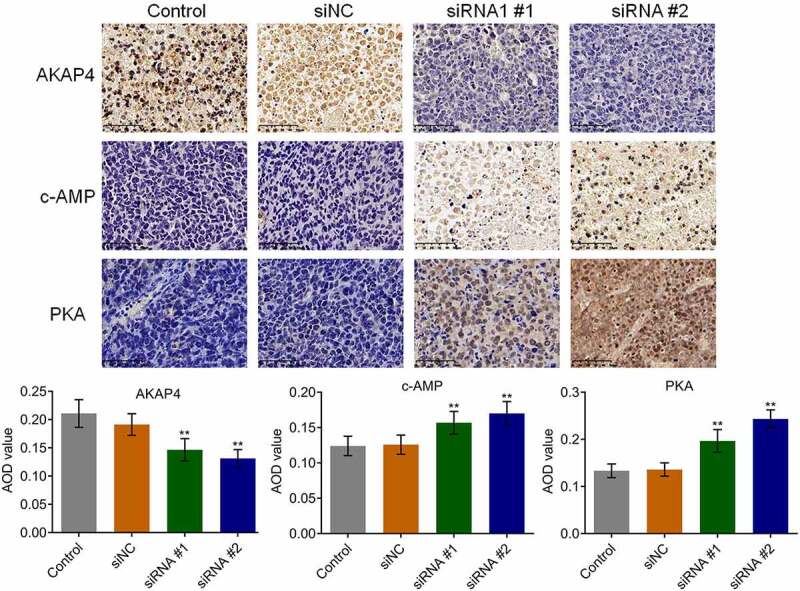
The c-AMP/PKA signaling in tumor tissues was activated by the knockdown of AKAP4. The expression level of AKAP4, c-AMP, and PKA was determined by immunohistochemical assay (**p < 0.01 vs. siNC). The controls were nude mice planted with H460 cells incubated with blank completed medium.

**Figure 7.**
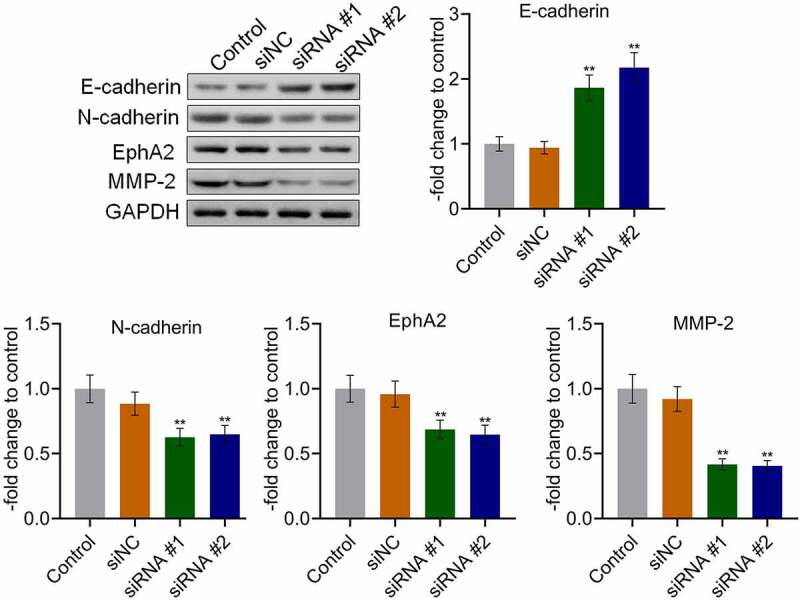
The EMT progression in tumor tissues was inhibited by the knockdown of AKAP4. The expression level of E-cadherin, N-cadherin, EphA2, and MMP-2 was determined by western blotting assay (**p < 0.01 vs. siNC). The controls were nude mice planted with H460 cells incubated with blank completed medium.

The state of the c-AMP/PKA pathway and EMT progression in the tumor tissues were subsequently investigated. As shown in Figure 6, compared to siNC, the expression level of AKAP4 was significantly decreased, and the expression levels of c-AMP and PKA were dramatically elevated in the siRNA #1 and siRNA #2 groups, respectively (**p < 0.01, vs. siNC), indicating an activation effect of the c-AMP/PKA pathway by the knockdown of AKAP4. In addition, compared to siNC, E-cadherin expression was greatly upregulated, and N-cadherin, EphA2, and MMP-2 (Figure 7) were significantly downregulated in the siRNA #1 and siRNA #2 groups, respectively (**p < 0.01, vs. siNC), revealing that the EMT progression in H460 xenograft tumor tissues was suppressed by the knockdown of AKAP4.

## Discussion

High expression of AKAP4 and the biofunction of AKAP4 in the development of malignant tumors have been widely reported. Recently, AKAP4 has been proven to be a promising diagnostic and prognostic biomarker for colorectal cancer [28], NSCLC [19], breast cancer [17], and ovarian serous carcinoma [14]. Jagadish reported that AKAP4 promoted the development and progression of colorectal cancer by regulating EMT [29]. Kumar claimed that the growth and survival of ovarian cancer cells were induced by AKAP4 through the regulation of c-AMP/PKA signaling [30]. However, the role of AKAP4 in NSCLC development has rarely been reported. In the present study, we found that AKAP4 was significantly highly expressed in clinical NSCLC tissues and NSCLC cell lines, which verified the conclusion by Gumireddy [19] that AKAP4 could be used as a potential biomarker for the diagnosis of NSCLC. We further knocked down the expression level of AKAP4 in H460 cells and significantly reduced cell viability, accompanied by increased apoptosis and decreased migration ability. Xenograft experiments verified the facilitation function of AKAP4 in the growth of H460 tumors. In addition, the *in vitro* and *in vivo* angiogenesis of HUVECs was suppressed by the knockdown of AKAP4. These functional experiments revealed that AKAP4 is a functional antigen that facilitates the development and progression of NSCLC.

Three domains have been reported to be involved in the structure of AKAP4, including a PKA binding domain, targeting sequence, and binding sites for other signaling molecules. AKAP4 binds to the R subunit of PKA through the PKA binding domain, leading PKA to its specific substrate for catalyzation. Recent reports have claimed that cAMP-PKA suppresses the proliferation, migration, and angiogenesis of malignant tumors [31,32]. In the present study, we found that the expression of PKA and production of c-AMP were negatively regulated by AKAP4 in both H460 cells and the H460 xenograft model, indicating that AKAP4 might promote the progression of NSCLC by regulating cAMP-PKA signaling, which might be related to the degradation of PKA induced by AKAP4 described previously [30].

Tumor migration is mediated by multiple elements, such as EMT, the tumor microenvironment, and angiogenesis. EMT is a progression of transformation from epithelial cells to mesenchymal cells under specific pathological states, contributing to the migration and distant invasion of tumor cells. EMT progression is commonly accompanied by the downregulation of epithelial markers (E-cadherin and keratins) and upregulation of mesenchymal markers (N-cadherin, EphA2, and MMPs) [33,34]. In the present study, we found that EMT progression in H460 cells and xenograft tumor tissues was significantly suppressed by the knockdown of AKAP4, indicating a positive regulatory effect of AKAP4 on EMT progression, which was consistent with the report described in colorectal cancer [29].

However, whether AKAP4 induced the development of NSCLC by regulating the cAMP-PKA pathway, EMT progression, or both needs to be further verified in our future work, such as the co-treatment of the inhibitor of the cAMP-PKA pathway or the activator of EMT progression in siRNA-AKAP4 transfected H460 cells. AKAP4 also binds to other signaling molecules (such as PKC, PP2B, PDE, and PP1) to induce interactions between PKA and these molecules. AKAP4 controls the activity of c-AMP/PKA signaling in the cytoplasm and nucleus via PKA-mediated phosphorylation and dephosphorylation by anchoring PKA to the appropriate site in cells [35,36]. In the future, we will explore more potential functional molecules that interact with AKAP4 to understand better the regulatory mechanism of AKAP4 in the development and processing of NSCLC.

## Conclusion

In conclusion, our study has revealed that AKAP4 might be an important target for treating NSCLC because of its function in promoting the migration and proliferation of NSCLC cells.

## Highlights

- AKAP4 facilitates the development of NSCLC;
- AKAP4 activates EMT progression;
- AKAP4 inactivates the c-AMP/PKA pathway.
